# The ethics of sense-making

**DOI:** 10.3389/fpsyg.2023.1240163

**Published:** 2023-12-08

**Authors:** Martin Weichold, Laura Candiotto

**Affiliations:** ^1^Institute of Philosophy, Technical University Dresden, Dresden, Germany; ^2^Center for Ethics, Department of Philosophy and Religious Studies, University of Pardubice, Pardubice, Czechia

**Keywords:** enactive ethics, sense-making, participatory sense-making, interpretation, world-making, self-making, eudaimonic well-being, mindfulness-based ethics

## Abstract

In this paper, we contribute to the arising field of “enactive ethics,” that is, the application of enactive cognitive science to the field of ethics. To this end, we will make a case that an “ethics of sense-making” should exist. With “sense-making,” we mean the permanent everyday embodied activity of interpreting the surroundings we are in, as well as our role in them. In other words, we mean the activity of understanding our environments in such a way that certain things, but not others, stand out as meaningful and relevant to us. We argue that sense-making can be performed in ethically better or worse ways. For example, one might make sense of a potentially provocative comment either as an insult or as an invitation for a respectful discussion. How one makes sense in this case will affect oneself, the other, and their present and future relations. We propose that it is often helpful to hold humans responsible for their ways of sense-making. This opens up the possibility to transform their sense-making and the worlds they inhabit. This also has significance for their eudaimonic well-being. Our ethics of sense-making focusses on the ubiquitous activities of sense-making, which, when changed, will lead to great ethical improvements of people’s actions, choices, and character traits.

## 1 Introduction

The realm of ethics has continuously been modified and extended. For instance, nowadays, it does not only concern intentional actions of human beings any more. Rather, it also encompasses animal ethics, robot ethics, ethics of beliefs, and ethics of unconscious biases. In this paper, we propose extending the realm of ethics even further: to ethics of sense-making. “Ethics of sense-making” denotes, we propose, a specific *topic*—namely sense-making—that should be investigated from an ethical point of view. By analogy, robot ethics investigates several ethically relevant questions concerning robots from an ethical point of view, and animal ethics investigates several ethically relevant questions concerning animals from an ethical point of view. Likewise, ethics of sense-making investigates ethically relevant questions concerning sense-making from an ethical point of view. As we will argue, ethics of sense-making is not just another further extension of ethics, but a particularly fundamental one. Ethics of sense-making is crucial for promoting and cultivating eudaimonic well-being, both at the individual and the social level. It can support the creation of shared values, and of better worlds to inhabit. It has the potential to be applied in many and different educative and therapeutic contexts. Since it relies on the natural capacity of human beings to make sense of themselves, others, and their social and cultural environments, it is also easier to promote, apply and follow than other ethics that need to employ external factors (such as rules and principles in deontology) to run against humans’ natural tendencies.

With “sense-making,” we mean the permanent everyday embodied activity of interpreting the surroundings we are in, as well as our role in them. In other words, we mean the activity of understanding our natural, social, and cultural environments in such a way that certain things, but not others, stand out as meaningful and relevant to us. To be clear, “sense-making” is the core concept of so called “enactive cognitive science.” We will come back to it in the due time, also stressing our contribution to the novel research line of so-called enactive ethics.

For now, let us convey our key message with an everyday example: if your neighbor tells you that he thinks that how you clean the communal stairs is completely useless—which meaning does his utterance have for you? Do you view it as an insult? Or as a provocation? Are you pretending not to hear? Or do you view it as an invitation for a respectful discussion? Or do you take it as an opportunity to check if your cleaning methods are efficient? The soundwaves that come out of the neighbor have no meaning in themselves—the meaning is generated in the relational interaction between you and your neighbor. It is imbued of the shared history of your previous interactions and is projected to the future interactions you will possibly have. Often, this meaning-generation, or sense-making, happens habitually and pre-reflectively, especially in its affective dimensions. Some people will immediately feel insulted, for example. However, our ethics of sense-making claims that we could and should not be passive bystanders to how our brain–body-systems continuously make sense of our surroundings. Rather, we can become mindful of how we are continuously making sense, and we can—in different, more or less complicated ways—improve it—whether to “heal” ourselves or to improve it in light of ethical considerations.^1^

In other words, sense-making can be performed in ethically better or worse ways. For instance, making sense of the utterance of the neighbor as an instigation for a heavy fight might lead to unnecessary suffering, in the present and the future. On the contrary, interpreting it as an invitation for a respectful discussion can be very beneficial in many respects. The easiest way to see this is to acknowledge that the way we make sense of our environment can lead to better or worse ethical consequences, such as joy or suffering. However, as it will become clear, it is not only the ethical consequences that matter. Making sense of our environment skillfully can itself be a virtue. And certain acts of sense-making—for instance, of other human beings as mere resources to be exploited—might be categorically wrong. It follows that acknowledging the pervasive impact of sense-making, and responsibly managing how we interpret the worlds we inhabit, has a significant ethical value. This is what an ethics of sense-making is all about.

In this paper, we will make a case that an ethics of sense-making should exist. It should exist as a topic, or sub-field, in ethics, like animal ethics or the ethics of belief. In this sense, our argument resembles early arguments that said that an animal ethics or an ethics of belief (Clifford, 1999) should exist. However, in this paper, we will not argue for a fixed ethical system that says exactly which ways of sense-making are the best ones. A fixed ethical system would be in contrast with what we take to be one core assumption of enactive ethics i.e., participatory sense-making, as we will explain in a moment.

There needs to be a new ethics of sense-making because standard approaches to ethics—such as consequentialism (Mill, 1861), deontology (Kant, 1785), and virtue ethics (Kegan, 1998)—do not explicitly account for sense-making. Consequentialism is focused on making choices (namely those with the best consequences); deontology on right actions (namely those that are allowed); and virtue ethics on building character traits (namely those that are virtuous). None of these ethical theories is explicitly focused on sense-making, even though hermeneutical thinking itself has a long history.^2^ By contrast, an ethics of sense-making emphasizes that the meaning, or sense or matter of significance, in a situation is always made, or brought about. According to our view, sense-making is a topic which consequentialism, deontology, and virtue ethics have completely overlooked, with their emphasizes on choices of consequences, actions, and character traits. Still, there can be consequentialist, deontological, and virtue ethical approaches to the topic of sense-making. This parallels other fields like animal ethics: the topic of animal ethics was new in the 1970s, but there are consequentialist, deontological, and virtue ethical (and many other) approaches to animal ethics. For instance, its focus on autonomous persons and their rights has made deontologists overlook the importance of animal ethics. However, now that the subfield of animal ethics exists, philosophers have even used resources from deontology to account for animal ethics (Korsgaard, 2018).

This paper has the sole intention to argue that the topic “ethics of sense-making” should exist. The aim of this paper is not to develop a full-fledged normative account that says which ways of sense-making are the best, or whether consequentialist or virtue ethical or more progressive approaches to the ethics of sense-making are the best ones. This paper does not aim to offer solutions, but rather to point to many “construction sites,” which future researchers of the ethics of sense-making are invited to work on. These include, among other things, the questions what ethically good sense-making looks like, how much control we have over our own sense-making, and how far we are allowed to intervene in the sense-making activities of others. In what follows, we will discuss these and many more new questions and challenges that come up once sense-making is investigated from an ethical point of view.

Against this background, this paper proceeds as follows: In section 2, we will present a variety of examples that should illustrate the importance and ubiquity of cases of ethically relevant sense-making. In section 3, we will dive deeper into the nature of sense-making. In particular, we will argue that an ethics of sense-making should heavily rely on insights into the nature of sense-making from so-called enactive cognitive science—while, reversely, enactive cognitive science shows its full relevance once it is applied to ethics. In this section, we also engage with and distinguish our approach from other accounts in the enactive literature. Against this background, in section 4, we will present a structured overview over different ethical issues in sense-making. In section 5, we will make some first tentative normative explorations for ethical values that should guide human sense-making. In section 6, we will discuss in which sense sense-making is up to us, and in which sense we are responsible for it. Finally, in section 7, we will conclude the paper by stressing the relevance of an ethics of sense-making to eudaimonic well-being.

## 2 Examples for the ethical relevance of sense-making

To start our illustration of an ethics of sense-making, in this section, we will present a variety of different cases of ethically relevant sense-making. We start with simple cases, although most real-life cases are more complex. We will say more about this complexity immediately after the presentation of the examples and throughout the paper. A more systematic overview will follow in section 4.

### 2.1 The perfect Ida

Imagine Ida. Based on how she has been educated by her parents, TV shows, and other influences, Ida has formed the attitude that everything in her life must always be perfect, and that she deserves it. Against this background, she judges harshly on everything and everyone in her environment. She always complains. She is always worried that her friends, partner, and job are not good enough. There is always something that Ida does not like about the situations she is in. Ida lives, so to speak, in a “complaining world.”

### 2.2 The radical Lea

Imagine Lea. She is very concerned about what she views as morality. In the course of the decades of her life, her moral views have become more radical. Every weekend, she participates in some sort of civil disobedience. She only meets with like-minded people, and breaks contact with anyone who does not share her views. Eventually, she is not even able to comprehend that other people have different views than she has. For her, everyone outside of her group is simply evil, corrupt, and ignorant.

### 2.3 The victim John

Imagine John. John is 40 years old, and from his point of view, everything in his life went wrong. For years, John was blaming himself, but eventually, he found a much better explanation of his fate: he is a victim. He is a victim of the larger social and political structures in which people of his kind simply have no chance of achieving anything. As a consequence, John stops blaming himself, but he lacks autonomy and feels heteronomous and disempowered. He gives up hope. He fails to see that there are many opportunities for improving his situation.

### 2.4 The greedy Lior

Imagine Lior. Lior thinks that life is all about getting what one wants. His entire thinking is focused on how he can realize his own wishes and desires. He views others only as means for getting what he wants. If another person suffers, this does not motivate him to help. To his own surprise, Lior feels very unhappy even though he is extremely successful in getting any single thing he wants. There are just a few—very rough—cases of ethically relevant sense-making. They show a diverse spectrum of cases in which sense-making matters to the lives we live. It matters to how we perceive ourselves, to how many and which problems we deal with, to how well we are faring, and to how caring and compassionate we respond to others. For now, the details of the different cases are not important. What we want to illustrate are merely the incredibly many facets in which sense-making impacts our lives.

### 2.5 Enlarging the scope

We want to add only one more point: in most cases, sense-making is a joint endeavor or, in technical jargon, participatory sense-making (De Jaegher and Di Paolo, 2017). Imagine Ida with her complaining attitude and John with his victim mentality together. Maybe they are a romantic couple. Then this could lead to even stronger, mutually enforcing dynamics: When their worlds melt together, Ida and John might constantly complain about everything, but see no way out of their misery, because they take themselves to be victims of unjust structures. These intersubjective dynamics are crucial: it is not the case that Ida’s perfectionism is simply her character trait. Ida interprets herself, John, their relationships, and the worlds they inhabit through the perspective of perfection. Perfection is the *how* of her sense-making, the specific flavor of her interpretation.

Now imagine that such problematic ways of sense-making are not only embraced by a romantic couple, but also by large social groups.^3^ Obviously, a lot of things in a society change once there are large groups in a society that take themselves to be powerless, but angry victims of unjust structures. And obviously, it changes the inner structure of a society tremendously if there are many people like the radical Lea—people who are so deeply immersed in their moral and political views that they cannot even comprehend that other people view things differently. This means that sense-making is always situated and its ethical impact goes beyond the sense-maker.

To see the social relevance of sense-making, imagine a particular situation in John’s life. John works as a policeman in Denmark. A few hours ago, he has found a refugee, and he has just finished interrogating him. As it turns out, the refugee aims at having an asylum status in Denmark. He is a political refugee and has no other place to go, but there are many of his relatives living in Denmark already. John concludes that the refugee has very good chances of gaining asylum in Denmark. However, just as the refugee is about to leave the police station, he mumbles that he has entered the European Union already in Greece. John could simply decide to overlook it. However, for John, correct rule-following is more important than anything else. He immediately enters the fact into the database, and the refugee gets deported to Greece.

Ways of sense-making can also lead to immoral behavior–for example, while radical Lea acts in the name of morality, she is only a few steps away from killing her political opponents. This highlights the practical need of an ethics of sense-making: sense-making is far from neutral and, although many times it gets habitual and automatic, agents can and should take responsibility for it. Not only for their own wellbeing, but also for the social good. We will come back to this topic in sections 6 and 7.

These cases illustrate the wide realm of cases of ethically relevant sense-making, from the ones that are more concerned with individual wellbeing to those that have a larger scope.

## 3 The nature of sense-making

What exactly is sense-making? Our answer has three parts. First, we start with a nearly trivial observation about the ubiquity of sense-making. Second, we continue with some “enactive insights” that follow if one thinks out the trivial observation. Third, we engage in some conceptual engineering and theory-building. This only serves for developing conceptual tools for talking about details of sense-making processes in the most helpful way. One does not necessarily have to buy into our specific conceptual proposal in order to reflect about the ethics of sense-making. However, we claim that this enactive approach to sense-making can offer important insights to the contemporary ethical discourse. At the same time, we stress that unveiling the ethical significance of sense-making and fostering its real-world’s applications is extremely beneficial to amending a merely descriptive account of it in enactive cognitive science.

### 3.1 Ubiquitous interpretation

First, let us begin with the trivial observation. Whenever we human beings experience and perceive something, we are interpreting. Now, whether or not this thesis is true depends, of course, on what is meant by “interpreting.” If it means a deliberate, self-conscious, effortful interpretation, the thesis would be false. Then, we would only be interpreting when trying to make sense of a complicated legal text or a novel from Kafka, but not when perceiving a traffic sign, for example. However, we understand “interpretation” in a much wider and pervasive sense. It means any process through which living organisms bring about meaning (Weichold, 2018; Weichold and Rucińska, 2022b). A piece of red and white iron in a triangular shape is not a traffic sign in itself. Its practical meaning—stop and give way—is dependent upon a culturally inherited road code.^4^ We might not know what a particular traffic sign in a far-away country means. A traffic sign here and now has only meaning for us. It has meaning for us because we are participants in a social practice where the traffic sign has this meaning. Also, it has meaning because it replies to our existential concerns, in this case the ones related to our and others’ safety: arguably, without traffic signs we will get easier injured while driving a car or riding a bike. In virtue of dispositions that we have acquired as participants in the practice, we are able to bring about the meaning of the traffic sign here and now. And this process is ubiquitous. A red leaf on the road has no meaning it itself. It can gain a meaning for us only if we make sense of it. Although the meaning is socially constituted, there are personal idiosyncrasies in how we make sense of something. We can make sense of it as beautiful, or as reminder of transience, or simply ignore it. And we can interpret it differently if, for example, a friend illuminates an aspect of the red leaf we overlooked. There is always a multitude of processes in human brain–body-systems that contributes to bringing about meaning in each moment—to making sense of the situations we are in.

These processes operate on many different levels. They encompass schemas and concepts, language, emotions, affective bonds, stress levels, unconscious biases, energy levels, habits, other kinds of dispositions, and many more factors. There are particularly rich dynamics once at least two organisms make sense of each other and each other’s sense-making, so that a complex form of “participatory sense-making” arises. In this sense, interpretation is ubiquitous in our life.

Now, this first point can be appreciated without having to buy into enactive cognitive science. We regard this as a virtue, because it means that one can appreciate the relevance of an ethics of sense-making without having to accept the enactive approach. However, the enactive approach is crucial for an ethics of sense-making because it highlights its existential significance. It follows that the enactive approach allows deepening the mentioned points in decisive ways.

### 3.2 Sense-making as core of existence

So, let us continue with our second point. It is false, or at least misleading, to say that certain events simply “have” meanings. Let us imagine perfect Ida’s friend forgets calling her back. From Ida’s perspective, this event is a scandal worth complaining about, as it shows that the friend does not treat Ida in the way she deserves. However, it now becomes obvious that the meaning exists only for Ida against the background of her complaining attitude. The friend has really forgotten to call her back, so what happened is not just her projection. But still, the particular meaning the event has for her is the result of sense-making. Meaning always exists in a relation. To understand sense-making, we need to focus on the relations between agents and their situations (Weichold, 2018). Relations come first. It would be a mistake to search for meaning only in objective surroundings or only in the minds of agents. Meaning is in-between. So, sense-making is a relational process of signification between agents and their situations. A further consequence is that meaning is always created in a continuously on-going process. One and the same agent might make different sense of the same event at different points in time. But this does not mean that whatever interpretation would have the same value. The specific interpretation would be out of what they care about and why they are significant for them. So, there is an existential question at the ground of sense-making, i.e., what is at stake for this agent in this moment? And different agents might make different sense of the same event. Sense-making is a permanently on-going process of relating to one’s situation, thereby transforming it and oneself from within.

Enactive cognitive science helps us in understanding sense-making in a more fundamental manner. This meaning creation we are talking about emerges out of existential concerns and constitutes the subjects and the environments they inhabit. Sense-making is embodied (Varela et al., 1991), namely as a process that is shared by all living systems^5^ and that speaks about their fundamental vulnerability and interdependence.^6^ Through living and engaging in a certain environment, organisms build themselves and transform their environments in order to reply to their fundamental needs and concerns.^7^ The environments, on their part, replies to sense-making and bounce back to the agent so that they change her too. Therefore, the relationship between agent and environment is circular, or in enactive terms, of mutual co-dependence.^8^

This is important to stress because, although sense-making for us humans is often performed in a conceptual-linguistic manner, sense-making is more basic and widespread. This is evident if we consider how much we make sense of a situation through emotions. Just think about how revelatory is Lior’s unhappiness or Ida’s dissatisfaction about how they find themselves in the world they inhabit.^9^ Sense-making is a situated and embodied practice.^10^ The disgust one’s feel when smelling a rotten meal reveals something of how one makes sense of the meal, namely as something dangerous and unhealthy. The joy one’s feel when meeting a friend after a long time signals how much the friend is dear to us. The anger that bursts out in facing an injustice reveals how much that particular situation and the people involved matter. It follows that sense-making is existential, not simply a projection or a subjective frame through which we perceive reality. As De Haan (2020: 126) put it, we are *existential sense-makers* because through sense-making we make a stance, we commit to a certain sort of action and way of inhabiting the world. This does not contradict our view of sense-making as pervasive interpretation, but it allows us to highlight its thicker, wider, and more constitutive character as core of existence.^11^

### 3.3 Some conceptual tools for thinking about sense-making

Third, let us propose some concepts for articulating aspects of sense-making we found through the examination of our examples. We start with *human organisms*.^12^ From a detached perspective, there are human organisms in *objective surroundings*. By definition, objective surroundings have no particular meaning to the organism. However, human organisms will quickly *make sense of their surroundings*. They will make sense of them, or enact, a *meaningful situation*. In other words, a meaningful situation comes about when a human organism *relates* to surroundings in a sense-making way. In enactive terms, *self and world are co-constitutive*. For example, Ida has enacted the objective surrounding of not having received a call from her friend as a meaningful situation in which her friend has treated her badly.

We propose analyzing meaningful situations in terms of *solicitations* and *affordances*. A *solicitation* is an invitation, or tendency, for acting, thinking, or feeling. In the way Ida brings about situations, there are many solicitations for feeling treated unjustly, and for complaining. *Affordances* are potential solicitations (see Weichold, 2018). Solicitations are based in the socio-material surroundings. But by definition, solicitations exist only in relation to human organisms.^13^

Meaning-making is an activity of human organisms that relate to their surroundings, and thus bring about meaningful situations with their solicitations. Human organisms are equipped with a variety of dispositions for making sense of their surroundings. One might call the more stable and deep dispositions “*enacting selves*” (Weichold, 2017). This has to be distinguished from the self-image, or self-interpretation that human organisms are continuously bringing about. This self-interpretation can be called *“enacted self”* (Weichold, 2017). For example, imagine the greedy Lior who always gets what he wants. He thinks he is the smartest person in the room. It is his enacted self, which he always brings about, that he is very successful. This is how he perceives himself. Now imagine further that IQ tests show that Lior’s IQ is actually below average level. Then, we can assume, Lior does not really have dispositions to say smart things, for example. However, he still has deeply engrained dispositions to constantly enact a self-image according to which he is the smartest person. These dispositions are part of his enacting self. They are part of the many deep, well-trained habits to make sense of himself and his environment for getting what he wants. The enacting self and the enacted self are the two ways through which sense-making is “*self-making*,” namely constitutive of personal identity.^14^

From moment to moment, human organisms make new sense. However, there are more *stable patterns*. These patterns can be based in *dispositions* and *habits* of human organisms.^15^ One example would be Ida’s deeply engrained habit of complaining about everything. In other words, the disposition to constantly complain about everything is part of Ida’s enacting self. Against this background, she constantly enacts a self-image, an enacted self, according to which she is a princess-like person whose needs, wishes, and cravings are all-important, while the world is a bad place because it does not give to her what she wants and (in her view) deserves.

### 3.4 World-making: the radical situatedness of sense-making

The mentioned patterns can also be based in features of the surroundings and interaction. For example, the way that John led the interrogation and the material arrangement and atmosphere of the interrogation room disposes the refuge to reveal the secret about his previous entrance in Europe. The patterns are often founded in social practices. One can call *“world-enactors”* such stable patterns of dispositions for bringing about more or less stable patterns of situations. And one might call “*a world*.” All the affordances that exist against the background of one set of world-enactors. For example, there is Ida’s world that is centers of complaints. There is the world of the Ida-John-couple. There is the world of Lea’s radical activist group. Once Lea starts feeling being a member of her radical activist group and is on the move with her fellow activists, she experiences a lot of solicitations for activism. She might decide to embark into a boycott campaign and thus enacts her *micro-world* of boycotting multinational products by buying Km zero products only and campaigning in front of big supermarkets to dissuade costumers to buy products there.^16^ This boycott world can last few weeks or become a more stable feature of her experience. But the point is that by focusing on agents’ dispositions and habits of sense-making it appears evident how much sense-making is *world(s)-making*, namely constitutive of their inhabited world(s).

Against this background, it is possible to distinguish between an *ethics of sense-making* and an *ethics of world-making*. “Ethics of sense-making” is the more general term and also encompasses an ethics of world-making. But an ethics of sense-making focusses also on spontaneous, unique sense-making processes that are not informed by stable patterns, or world-enactors. By contrast, an ethics of world-making focusses in particular on the ethical evaluation of worlds that are created. Is it possible to flourish in a complaining world? Is it beneficial for democracy if large parts of the population live in a “victim world”? An ethics of world-making does not only focus on the enacting selves of individual human organisms, but on all kinds of world-enactors, such as the design of social practices and social institutions, and the make-up of socio-material surroundings.

To say that an ethics of world-making is a sub-species of an ethics of sense-making does not dismiss its value. On the contrary, it allows us to focus even more on the implications of the radical situatedness of sense-making. Also, focusing on the ethics of world-making helps us to avoid any possible individualistic residue of an ethics of sense-making. To clarify it, imagine Maria.

Maria is a lesbian Hispanic person living in the United States of the 1970s, after having immigrated from Argentina. She has to live with the fact that nearly no one shares her particular world view. Her mother in Argentina does not have any experience about the United States, many of her friends in the United States not share her particular background, and so on. Moreover, Maria has the impression that others always “aggressively” project their particular world’s views, values and expectations on her, and judge her accordingly. Consequently, Maria feels that she constantly has to “travel” between different worlds, while it only rarely happens that people try to understand her own perspective.^17^

The case of Maria is far from unique: very often radical embeddedness comes with incommunicability and the incapacity of making-sense of others’ sense-making. This means that although sense-making is participatory from the beginning, this does not imply that sense-makers inhabit a shared world with shared meanings and values by default. A shared world might be a goal, but it is never a starting point. For getting to it, Lugones (1987) recommend “*worlds traveling*,” that is inhabiting different worlds with openness, curiosity, and receptivity, exploring different ways of sense-making, but without the obligation to become native to the others’ worlds, thus preserving one own’s world. Worlds traveling also implies being able to accept to not always understand another person, a situation, an event and even yourself,^18^ to live with the dissonance of non-sense (Vörös, 2017). But worlds traveling could dismantle the arrogant perception that we described in the case of the radical Lea. It might even imply to accept, instead of fight, contrastive cultural values. This is important to stress because we advise to take situatedness in an ethics of world(s)-making in a pluralistic manner so that we can avoid the traps of parochialism.

Also, it points to the necessity of dismantling certain detrimental worlds. This means that an ethics of sense-making also comprises *world-breaking*. Sense-making is not just world-making. Actually, in certain situations, most notably in the therapeutic context, breaking a sedimented detrimental meaning of oneself and of a situation is crucial for achieving a new and a better process of self- and world-making.^19^ For example, by interacting with John, we might need to transgress his boundaries (Maclaren, 2018), and help him in destroying its victim world.

## 4 Ethical issues in sense-making

As it has hopefully become clear, sense-making is a complex process with a multitude of aspects. An ethics of sense-making can make recommendations about all of those different aspects. In this sense, it does not have simply one target, but many. Let us present an overview of the different aspects that an ethics of sense-making can evaluate.

First, an ethics of sense-making can evaluate the enacting selves of individual human beings, that is, their *dispositions* for making sense. An ethics of sense-making resembles virtue ethics in this sense. Among other things, it can evaluate whether the interpretations of individual persons are really helpful. For instance, it is really helpful that Lior thinks that life is only about getting what one wants, or that John thinks he is a powerless victim of unjust social structures?^20^

Second, an ethics of sense-making can target *particular processes* of sense-making. Is the process of participatory sense-making between John and the refugee a good one? As further examples, one might also think of very hierarchical interactions. Is the participatory sense-making that emerges from such instances of participatory sense-making really helpful? In this sense, an ethics of sense-making evaluates something that no other (Western^21^) ethics evaluates: namely specific *relations* between (human) beings—and not just character traits, intentions, or consequences.^22^

Third, an ethics of sense-making can become an ethics of *world-making*, and evaluate the worlds that individual human beings or larger groups of human beings bring about. Is Ida’s “complaining world” really helpful? Cana society where large groups of people take themselves to be powerless victims be a flourishing society? An ethics of world-making can target different world-enactors. It can target the dispositions of individuals for bringing those worlds about (as described in the first point). But it can also target dispositions (affordances) in the socio-material environment for bringing certain worlds about. And it can target respective social practices. In this sense, an ethics of world-making overlaps with an ethics of designing social institutions (Jaeggi, 2014).

Evaluating sense-making in these three regards is no easy task. There are many kinds of conflicts, tensions, and problems involved. For example, what is a good interpretation of life in the first place? John’s victim’s mentality has both advantages and disadvantages. For instance, it has the advantage that it allows John to stop blaming himself, but the disadvantage that it disempowers him. Or, to consider another example: A Stoic attitude of deeply accepting one’s fate might lead to inner freedom and inner peace. But at least at first sight, it has the costs that one might not fight for valuable projects anymore, e.g., for more social justice, empowered by a feeling of justified rage. Is there a more balanced way of utilizing deep ideas – such as the idea of universal acceptance – without becoming completely absorbed by them?

Another problem is that it is not clear how a good instance of participatory sense-making should look like. Imagine we meet Maria. Then it seems helpful to engage with her perspective and her ways of making sense, instead of projecting our standards on her. But should this always be done? Imagine we meet Lior, who is so deeply absorbed in his world of “getting what he wants,” suffers from it, but cannot get out of it. Is it really the best choice to engage with his way of sense-making deeply? Or should we better try to intervene and help him out of his misery? However, very likely he would find any attempt of intervention intrusive, and view it as an instance of arrogant perception (Lugones, 1987) where one project one’s own values on others’ life. So, should we respect Lior’s autonomy more than our possibility for helping him to stop suffering from his own way of sense-making? Or, consider Lea. She is completely absorbed in her radical activist world so that she cannot see any other values than her own. This is problematic, but at least in the present moment, she does not even see that there is any issue with it. Indeed, she is completely unaware that she has brought about a rather narrow and limited world. For her, her world simply expresses “the moral truth.” It is not even the case that she does not reflect—but all her reflection is framed by and takes place in her radical activist world. Imagine she does not only suffer herself from her way of sense-making, and loses all her friends who do not share her views, but that she starts violating ethical norms and using violence against her political opponents. Is there a point at which her sense-making becomes morally problematic?

Or, consider an entire society. What happens to a society if it consists of more and more different groups with their own worlds, such that the groups do not even understand each other’s worlds anymore? Is this in the spirit of pluralism? Or, has there at least to be some common ground, some minimal consensus? If so, how could it be achieved, given that it has already been lost?

These are just some of the problems that an ethics of sense-making points to. To our mind, these problems are not problems *of* an ethics of sense-making. Rather, these problems permeate our daily life, and it is a merit of an ethics of sense-making that it makes them visible and pushes to address them from within. In the following section, we will explain how an ethics of sense-making can do so, at least in a rough outline.

## 5 Ethical values for evaluating sense-making

A core question for an ethics of sense-making is: What are the normative criteria for evaluating sense-making? What is the axiological basis for judging that a particular instance or way of sense-making is better or worse than another one?

If our ethics of sense-making would be a Universalist religion, there would be a straightforward answer. Many religions come with a clear view of what the good and moral life consists in, and come with a clear set of principles. However, such religions are often experienced as maximally intrusive. They prescribe their views of a good life to everyone in a Universalist manner, without taking into serious account individual preferences and contextual differences. Given that an ethics of sense-making should be an ethics for each of us as embedded in specific life contexts and motivated by personal existential needs and concerns, and given that we are living in a pluralistic society with a great variety of conceptions of what life and what morality is all about, an ethics of sense-making cannot work like a universalist religion. Yet, it is possible for an ethics of sense-making to evaluate sense-making without endorsing Universalist claims and, so, without imposing a fixed view of the good life on everyone else. To explain this, we wish to make three points.

First, an ethics of sense-making has to be understood in terms of practice-oriented recommendations for those who are interested. To our mind, ethics is not about making strict prohibitions to others in a moralizing fashion. Such a “moralizing morality” only leads to conflicts and bad feelings, and does not lead people to change their behavior anyhow. Instead, our ethics of sense-making is about helping people to taking part to the ethical enterprise when they are interested. And the interested parties need not necessarily be individuals. They can, for instance, also be policy designers and designers of institutions. Even if, for instance, Lea might not be interested in hearing about the problems of her getting lost in her radical activist world, designers of institutions might be interested in changing world-enactors in such a way that not so many people get lost in radical activist, fundamentalist and extremist worlds.

Second, an ethics of sense-making can, in many cases, start from the values of the people involved. Even though most people have different accounts of what matters in life, they are human organisms at root. As such, they have existential needs. They need to find ways of living in which their existential needs and concerns are listened to and met at least to a sufficient degree. An ethics of sense-making can make this visible to people. In such a way, people can first become ambivalent about their problematic ways of sense-making, and eventually change them. For example, Lior is unhappy, even though his life is all about getting what he wants. There is something wrong with his way of sense-making by his own standards. An ethics of sense-making can make him realize this, without imposing specific values. It provides ethical analyses of the potentials and costs of people’s ways of sense-making by their own standards.

Third, there are still some external values that can guide an ethics of sense-making. We think that these values can be agreed upon by most people. We propose a set of specific values. However, these values are not part of a fixed conception of the good life that should then be imposed on others. Instead, our values are about *empowering* people to become freer and more autonomous in sense-making. Their nature is orientative and general enough to be carved out in answering to specific cultures, situations, and individual needs. This means that they will take specific shapes according to the moral agents and the situation they are in. Also, they are intended to be an ever-evolving resource for embodying an ethics of sense-making, being out of a commitment to the living and dynamic nature of what it is to work. However, a *file-rouge* can be found in them, namely the motivation to deal with sense-making responsibly, as we will see in the next section. We offer them because many people are not aware of their sense-making as sense-making. They are not aware that there are alternative ways of sense-making. They are not aware that it is a natural fact that different people make sense in different ways. Bringing this to their awareness has a heavy emancipatory potential. So, an ethics of sense-making can take an ameliorative shape, especially if endorsed along with educative and therapeutic interventions. In this sense, an ethics of sense-making can help people to liberate themselves from the worlds they have locked themselves in. It can help them to see more options for sense-making, to choose better ways of sense-making according to their own standards, to feel that they can do it and, so, become freer and more empowered.

So, we propose that an ethics of sense-making should, among other things, be guided by the following values:

### 5.1 Mindfulness

Sense-makers should be aware of their sense-making as sense-making. They must not be lost in or absorbed by their world. They should understand that the way they perceive things is, in part, due to their own way of sense-making in this particular moment. They can then pay attention to their sense-making practices, in the very moment they unfold (as a practice of intelligent awareness) and diachronically, as a constant reflection on their sense-making. For example, Lea lacks mindfulness by being captivated by her radical activist world.

### 5.2 Inner freedom

Sense-makers should not be in the grip of a particular way of sense-making. They should not be overwhelmed by thoughts or feelings that are enacted in a specific world. Instead, they should be able to have a distanced, detached attitude to their own sense-making. Correctly understood, inner freedom does not reduce one’s capacity to immerse oneself deeply into one’s situation, but enhances it (pace Dreyfus). For example, Ida lacks inner freedom because she is constantly overwhelmed by feelings of dissatisfaction that emerge in her complaining world.

### 5.3 Autonomy

Sense-makers should be able to be aware of how they make sense of their surroundings, and how they create worlds. This of course does not mean that they will have full-control over the situation. Or, that they would not be susceptible to the environmental conditioning and their personal and collective history. As we stressed, sense-making is always relational and sense-makers and their worlds are co-constituted. However, agents are not destined to be lost in specific worlds. They can become aware of how they make their worlds in each moment. This means that they can respond mindfully to the situations they are in, instead of reacting habitually. They can be aware of alternative ways to make sense of their present surroundings and, when needed, to change them. For example, Lior seems to lack autonomy, because he seems unable to get out of his “getting what I want”—world.

### 5.4 Openness

Sense-makers should be sensitive to others’ ways of sense-making and be ready to adjust their sense-making for replying to the others’ concerns. Also, they should be open to extend and enrich their and the others’ worlds through “worlds-traveling.” Consider again the radically activist Lea. Her worlds are not continuously getting richer and more interesting. She does not constantly “couple” her ways of sense-making with the ways of sense-making of others, such that new meanings emerge. Instead, by becoming more and more radical, what is meaningful to her—her own world—constantly becomes smaller and smaller.

### 5.5 Selflessness

Sense-makers should liberate themselves from the idea that there is such a thing as a true, authentic self. Instead, they should realize that their enacting selves consists of a variety of dispositions for bringing about different interpretations of themselves in different moments, without any interpretation being the sole and only “true” one.^23^ Against this background, they can enact different self-conceptions at different times, while remaining aware that these are mere conceptions (Weichold, 2017).

### 5.6 Agency

One value for selecting one way of sense-making over another is flourishing: Particular ways of sense-making can empower agents, while other ways disempower them. For example, John’s victim mentality makes him miserable and disempowers him from even trying to improve his situation. Even if there are really unjust social structures that have contributed to causing John’s situation, a self-interpretation according to which John is a responsible agent who can change the relationships he has with himself, the others and the worlds he inhabits might be much more helpful for him.

Let us finally say a word on *Truth*: Classical Virtue epistemologists are, to some degree, interested in similar points as an ethics of sense-making, seen as a specific instance of an ethics of knowing.^24^ However, their guiding values are first and foremost truth and knowledge. From the perspective of an ethics of sense-making, truth and knowledge matter. But they are often overrated (*cf.* Elgin, 2017). As it will become apparent in the section 7, the good life should not only be there too, but it is the existential ground of an ethics of sense-making. Consider John again. It is not helpful for improving his situation to get obsessed in finding out to which degree social structures are the cause of his fate. Of course, it is crucial to come to a full recognition of the determining effects of social systems in his life, but this should be used as the basis for empowered action. For doing so, a new self-interpretation of himself as a sovereign, responsible agent might be necessary. This self-interpretation might contain many fictional elements, though. Its aim is not to detect John’s true self, but to empower him to new agency.^25^

In sum, an ethics of sense-making evaluates particular instances and general ways of sense-making. Its criteria are the values of the sense-makers themselves, as well the just displayed values of mindful, free, autonomous, open, selfless and empowering sense-making. In this way, an ethics of sense-making does not dictate human beings in a universalistic moralizing fashion how they should make sense. Instead, an ethics of sense-making helps interested sense-makers to become clear about the practical and in particular the ethical effects of their ways of sense-making. It empowers human beings to take ownership over their own sense-making, and in realizing that they are contributing to the sense they make, helping them to assess whether the tradeoffs they are making are good. So, an ethics of sense-making can analyze the benefits and the costs of particular ways of sense-making. For instance, it can point out to John that even though his victim mentality brings him comfort, it comes with high costs. It can point out to Lea that even though she cares about morality, her behavior will have bad moral consequences, and that she is far away from being a moral role-model for others. In the end, an ethics of sense-making views John and Lea as autonomous persons: it is up to them to decide what they want to do once the potentials and costs of their ways of sense-making have been analyzed. An ethics of sense-making unfolds opportunities to take part to the ethical discourse in a participatory manner; it can offer advice and guidance, but no commandments. It empowers agents in taking responsibility over their sense-making practices.

It follows that an ethics of sense-making does not have to stop at its core business of analyzing the ethical potentials and costs of different ways of sense-making. It can also encourage individual human beings to become good sense-makers themselves. In this sense, an ethics of sense-making resembles virtue ethics. The just discussed values of mindfulness, inner freedom, autonomy, openness, selflessness, and agency can also be understood as virtues, or competencies, of ideal sense-makers. But the emphasis of an ethics of sense-making on *practices* (*cf.* Weichold and Rucińska, 2022a), especially in its enactive framework, makes it more practice-oriented than virtue ethics that is primarily focused on self-betterment. Understanding sense-making as a practice can then help solve the application problem of a *theory* of ethics. In the ethics of sense-making, questions such as “How should I apply this recommendation to my case?” are replaced by the practice of making sense of a situation, oneself, and the others by being there and actively taking responsibility for it. It follows that agents should not *apply* an ethics of sense-making, but engage with the situations as good sense-makers.^26^

As we said in the beginning of this paper, this is, of course, not yet a full-fledged ethics of sense-making, but rather an argument in favor of its existence. Among other things, a full-fledged ethics of sense-making has to find ways of dealing with the ethical issues mentioned in section 5—but this is beyond the scope of this paper. Our concern in this paper is not to develop a full-fledged ethics of sense-making, but to argue that an ethics of sense-making should exist.

## 6 Responsibility for sense-making

Are human beings responsible for their sense-making? From the point of view of our ethics of sense-making, there is a mistake in the question. The question presupposes that there is a fact of matter concerning whether or not human beings are responsible for their sense-making. Behind the question lies the objectivist metaphysics of agency that informs deontology. By contrast, our ethics of sense-making holds that it is a matter of sense-making whether or not the processes of sense-making themselves are individuated as responsible actions. Then, the crucial question is whether it is *fruitful or not fruitful to make sense* of a human being in a current situation in such a way that she is responsible for particular behavior. Our ethics of sense-making suggests that it is *fruitful* to *understand* human beings as being responsible for their sense-making. This is an interpretation with great potential (and some costs that have to be attenuated by a balanced usage of interpretation). The potential is that we humans can become much better at sense-making when we take responsibility for it. Then, we can profit from the advantages of our henceforth better ways of sense-making. Accordingly, an ethics of sense-making should encourage and empower human beings to take responsibility for their sense-making.

It might be also argued that there is an existential necessity to take responsibility for our and others’ processes of sense-making. The reason is that it would be very hard to inhabit a social world where there is no responsibility for how we make sense of that world, of ourselves and the others. At this regard it is important to stress once again that sense-making is a participatory practice that grounds human sociality and constitutes the social worlds we inhabit. Also, it is important to highlight that through shared practices of sense-making humans co-create the values that constitute the worlds they inhabit. Therefore, granting humans the responsibility on sense-making empowers them also from a civic and political perspective.

Taking responsibility for one’s sense-making is not easy. The reason is that sense-making is not completely under conscious control. As discussed earlier, there are many factors that contribute to how human beings make sense in a given moment: acquired habits, schemas, concepts, and frames, emotional and affective patterns, bodily background conditions, stress and energy levels, expectations, met or unmet basic needs in a given moment, and many more factors, including a rich variety of situational factors. Moreover, when there are two or more human beings involved who make sense of each other, complex dynamics emerge. Sense-making is a process that does not fit the traditional dichotomy of activity and passivity.

Still, in order to take responsibility for one’s sense-making, it is helpful to view it more as an activity. Indeed, there are many ways of how we can influence our sense-making, either directly or indirectly. At a given moment, one can try to become mindful of one’s sense-making.^27^ On can try distance oneself from the thoughts and emotions one has in a particular moment, instead of identifying with them and acting on the interpretation that automatically pops into one’s mind. Then, one can act on what happens in a more deliberate way, instead of reacting immediately. Before entering a situation, one can reflect on how helpful the expectations and schemas are with which one enters the situation. In the long run, one can try to change one’s general habits for sense-making. If sense-making is shaped by social practices, as we argue, it is diachronically extended and can thus be improved through training. For instance, this can be done by learning mindfulness meditation, attending cognitive behavioral therapy, or learning techniques of non-violent communication. But as described earlier, sense-making is a process of relating, and not a one-sided projection. One might also improve the socio-material side of the relation. Designers of behavioral settings, social institutions, and policies can intentionally change world-enactors. For example, a teacher can design a better working atmosphere in the classroom (Candiotto, 2019). Moreover, social practices come about by a constant repetition of particular interactions. As Butler (1991) has argued, these repetitions can intentionally be performed “falsely,” so that social change emerges. This is one possibility for changing the ways of sense-making that happen in given social practices in a more pluralistic and open framework.

Changing one’s ways of sense-making is often helpful from an ethical point of view – but in certain situations, changing one’s ways of sense-making can even be necessary. We are thinking in particular of those situations in which a specific type of sense-making is detrimental for the agent. Arguably, one of the main aims of a psychotherapeutic intervention is to enable the patient to change the detrimental sense-making into a new and more helpful way of understanding oneself and the relationship with the inhabited world. This change can be done in different manners, for example through dialogue, active listening, bodywork, art-therapy, etc.^28^ But the common trait is acknowledging that a process of sense-making is detrimental and working for changing it to a better one.

In sum, while sense-making is never fully under one’s conscious control, there are many possibilities for influencing it either directly or indirectly. Interpreting ourselves as autonomous human agents capable of responsible agency opens one up to these possibilities.

## 7 Eudaimonic well-being

We would like to conclude our argument in favor of an ethics of sense-making by stressing the relevance of sense-making to eudaimonic well-being. Differently from hedonic well-being, eudaimonic well-being does not rely on a feeling of satisfaction or happiness. On the contrary, it is rooted in the good life and it is achieved when the agent finds to live a life that is in balance with her values, and when her activities realize her aims.^29^ The life is then meaningful and worth living.^30^ Therefore, ethical sense-making appears to be crucial for eudaimonic well-being since it is through the enactment of meaning that oneself finds eudaimonic well-being and fights nihilism.^31^ Although being objective, this theory of well-being is agentive: the meaning of life is not just out there, the agent contributes to it and realizes herself through it. In enactive jargon, we can say that the agent embodies the meaning, with all the affective states that come with it. Moreover, as it has been stressed by Bishop (2014), positive emotions and attitudes contribute to well-being. The felt experience of meaning can thus play an important motivational role for fostering more stable habits of well-being.

However, as we stressed all along the paper, not all meanings are beneficial to the agents and/or her surroundings. Certain meanings are detrimental and need to be amended or even destroyed. That is why an ethics of sense-making is required. Firstly, it stresses this basic point: sense-making is not neutral. On the contrary, it is normative. There are sense-making activities that are better and other that are worse, both regarding the agents and the worlds they enact. This is our fundamental message to enactive cognitive science: the normative dimension of sense-making should be taken seriously and, thus, an ethics of sense-making is needed.^32^ Secondly, although an ethics of sense-making, as conceived by us, does not provide fixed regulations and standards for assessing sense-making, the focus on intelligent awareness of our own and others’ sense-making has strong ameliorative outcomes., To conclude, our ethics of sense-making focuses on the ubiquitous practices of sense-making, which, when revised an changed if detrimental, will lead to eudaimonic well-being. This means that if the practice-oriented recommendations we put forth are seriously discussed and implemented in different educative and therapeutical contexts, an ethics of sense-making can fruitfully contribute to the good life.

## Data availability statement

The original contributions presented in the study are included in the article/supplementary material, further inquiries can be directed to the corresponding author.

## Author contributions

All authors listed have made a substantial, direct, and intellectual contribution to the work and approved it for publication.

## Footnotes

^1^See section 3.2 where we go deeper into the enactive approach to sense-making as existential stance and section 7 where we depict the contours of responsible sense-making. Although we cannot develop it here, this has important therapeutic implication. We briefly introduce it in section 8 in relation to eudaimonic well-being.

^2^For a rich and important dialogue between enaction and hermeneutics about sense-making, see Dierckxsens and Bergmann (2022).

^3^It is important to stress that participatory sense-making is not only about dyadic interaction. On the contrary, participatory sense-making can be applied to a larger scale, and in this manner, it can be a powerful hermeneutical tool for tackling issues of social injustice, for example. See on this De Jaegher (2013).

^4^To be sure, we can imagine a traffic sign from an earlier culture, where the traffic sign is now “dead” because the social practice that gave life to it is gone.

^5^This is the continuity thesis between life and mind for which sense-making unfolds from processes of living. See on this Thompson (2007).

^6^This thick concept of “embodiment,” with an important existentialist flavor, also includes a phenomenological understanding of the living body, especially in its feeling dimension. See on this Colombetti (2017).

^7^At this regard, see the evolutionary biology’ work on niche-construction and its employment in ecological psychology and cognitive science regarding cognitive niches construction. See Clark (1998) and Chemero (2009).

^8^See Weichold (2015). In enaction theory, this has an important reference to Buddhist ontology and epistemology. For a recent debate about this, especially regarding its ethical implications, see Candiotto (2022b).

^9^On the revelatory aspect of emotions, see Solomon (1976) and Slaby (2008).

^10^For a theoretical and practice-oriented examination of meaning as embodied, see Schoeller (2022).

^11^This also stresses the difference between an ethics of sense-making and several psychotherapeutic orientations and interventions, although there might be important overlaps, especially with the existentialist tradition in psychotherapy. Also, as pointed out by one of our reviewers, the ethics of sense-making can sound similar to Cognitive Behavioral Therapy (CBT) since it puts emphasis on patients becoming aware and changing automatic thought-feeling processes. However, the enactive approach that is at the ground of our ethics of sense-making understands awareness and feelings from the perspective of embodied cognitive science and so, the responsibility for our sense-making is not reducible to a cognitive-linguistic practice. Moreover, as we will make explicit in section 7, the ethics of sense-making implies an ethical stance: people should change their ways of sense-making not only to improve their individual coping, but to realize ethical values.

^12^We limit our investigation to human organisms since our main goal in this paper is to shed light on the ethics of *human sense-making*. However, as already mentioned, sense-making belongs to every organism in enactive terms (see on this, Weber, 2016,^2019^). This is important to stress because the ethics of sense-making we are presenting here could be significantly applied in animal and environmental ethics by extending participatory sense-making to the living organisms and the ecosystems they inhabit (Candiotto, 2022b), also focusing on the “intrinsic value” of nature as the bedrock of environmental ethics (Werner and Kiełkowicz-Werner, 2022). For developing this research further, a non-anthropocentric understanding of “interpretation” is needed (*cf.* Weichold, 2018).

^13^On a relational understanding of affordances, see Rietveld and Kiverstein (2014).

^14^This shares some features with the “narrative self” approach. See on this Hutto (2016) and Piredda and Candiotto (2019).

^15^See Candiotto and Dreon (2021) and Weichold (2015) for accounts of habits that are resonant with the perspective we are developing here.

^16^“Micro worlds” is a notion that has been put forth by Varela (1999) in the context of ethical know-how. It refers to the enactment of specific and temporally determined worlds through sense-making activities. Their scope could have different sizes and comprise less or more interlocutors. This resonates with a more recent approach to “little worlds” as embedded in forms of living (von Maur, 2021).

^17^This case follows an autobiographical paper from Lugones (1987).

^18^This is very much in line with the key message of “loving epistemology” (De Jaegher, 2019).

^19^This is a crucial practice in existential psychotherapy and the most recent enactive psychiatry. See on this Stanghellini and Rosfort (2013) and De Haan (2020).

^20^What do we mean with “helpful” here? Is “helpfulness” a core value of an ethics of sense-making? As we understand it, our way of talking about helpfulness does not refer to a particular value. Instead, “helpfulness” is a *place-holder* for particular values. For example, a particular way of sense-making can be helpful to realize the personal values of the involved persons. Or, it can be helpful to reduce suffering. It can be helpful to enact eudaimonia. It can be helpful for the functioning of society. Which value is the crucial one? We want to leave this question open at this point. Different researchers on the ethics of sense-making can disagree and argue about this question. There can be eudemonic, consequentialist, deontological, pluralistic and many other varieties of ethicists of sense-making. Still, they would all share the idea that ways of sense-making are not God-given, nor that they are only to be evaluated according to the truth of their content. Instead, what is crucial is whether the ways of sense-making are helpful for realizing ethical values, whatever these values are.

^21^See Garfield’s comparative work (Western philosophy and Indian Buddhist Philosophy) for an ethics of interdependence (Garfield, 2022). Also, some recent investigations into Ubuntu ethics (Ujomudike, 2016), the ethics of the commons (Weber, 2020), and indigenous epistemologies could be very helpful for an ethics of sense-making.

^22^An ethics of participatory sense-making has been proposed by Candiotto and De Jaegher (2021) with a strong emphasis on loving the other in her difference as the core ethical practice. Our proposal can provide a more general framework for thinking about the ethics of participatory sense-making, also enriching the list and the analysis of the dispositions required to undertake participatory sense-making in an ethical manner.

^23^See again on this Garfield (2022) and Varela (1999).

^24^See, for instance, Axtell (2000).

^25^Although we cannot develop here the connections between the enactive and the pragmatist epistemologies, especially regarding the concept of truth (as in this paragraph) and the epistemic value of a community of inquiry (that is very relevant to the concept of participatory sense-making, see on this Candiotto, 2022a), we would like to flag the pragmatist flavor of this paragraph to draw attention to the practice-oriented approach and ameliorative aims of our ethics of sense-making.

^26^Di Paolo and De Jaegher (2021) have recently stressed that enaction is not a *theory* of ethics because ethical questions cannot be exhausted theoretically. We join their effort to stress the situatedness of ethical engagement. However, our ethics of sense-making shows that enaction is intrinsically ethical because sense-making is not a neutral practice but is what constitutes ourselves and the situation in a specific manner. Therefore, we claim, that our ethics of sense-making is more fundamental than a mere application of a theory because the boundaries between theory and application are replaced by the fundamental existential practice of sense-making. Still, a theory, if not dualistically understood as separated by its application, can be very useful and emerge out of sense-making.

^27^For a Husserl inspired enactive account of what does it mean and how to become aware, see Depraz et al. (2003).

^28^For an enactive approach on how to promote well-being in dyadic interactions, also within a therapeutic context, see Kyselo and Tschacher (2014) and García (2021). For an enactive approach to mental disorders, see García and Arandia (2022).

^29^To be clear, this does not mean that one must focus on the realization of one’s “true self.” As one alternative, some enactivists have proposed that one should focus on the process of becoming “selfless selves.” See on this Varela (1991,1999),Weber and Varela (2002), and Di Paolo (2021).

^30^Eudaimonic well-being has an ancient and noble breed (Aristotle’s ethics; see on this Kraut, 2007), but it has been advanced also in the contemporary debate as an excellent candidate to contrast the subjectivist theories of well-being (for the debate, see Fletcher, 2016). It has been applied in different sectors, from mental health to occupational theory and practice. See Hayward and Taylor (2011).

^31^Albert Camus wrote important pages on the dangerous effects of nihilism, as the incapacity to find meaning in life, to well-being. At the same time, he stressed the perils of semantic realism and thus argued for what we would define (although without the possibility to carefully engaging with his writings here) an enactive account of meaning. See Camus (1942).

^32^We are not alone in this call. Notably, Ezequiel Di Paolo, Elena Cuffari and Hanne De Jaegher, among others, have strongly advocated for the non-neutrality of the epistemic attitudes of the enactive program. See Di Paolo et al. (2018), ch. 12.
